# The effectiveness of using interactive multimodality books in pediatric nursing in implementing Merdeka Belajar program

**DOI:** 10.1590/0034-7167-2022-0599

**Published:** 2023-11-10

**Authors:** Sapariah Anggraini, Selly Kresna Dewi

**Affiliations:** I Institute of Health Science Suaka Insan. Banjarmasin, South Borneo, Indonesia

**Keywords:** Digital Book, Pediatric Nursing, Nursing Students, Learning, Curriculum, Livro Digital, Enfermagem Pediátrica, Estudantes de Enfermagem, Aprendizado, Currículo, Libro Digital, Enfermería Pediátrica, Estudiantes de Enfermería, Aprendizaje, Currículum

## Abstract

**Objectives:**

to analyzing the Effectiveness of Using Interactive Multimodality Books in Pediatric Nursing on Student Learning Outcomes in Implementing the Merdeka Belajar Program.

**Methods:**

the research design used a quasi-experimental approach with a posttest control group design. The sample in this study was the fourth-semester nursing students of Institute of Health Science Suaka Insan Banjarmasin who received the Pediatric Nursing course, a total of 52 students. The sampling technique used is total sampling. Data analysis used the dependent t-test.

**Results:**

the interactive multimodality pediatric nursing book was effective in improving the learning achievement of fourth-semester students with a significance value of 0.015 < 0.05. There is a difference in the average value of pediatric nursing courses for class A and class B, which is 3.173.

**Conclusions:**

the use of interactive multimodality books is very good for lecturers to apply in teaching to help students understand the material they are learning.

## RESUMO

**Objetivos:**

analisar a eficácia do uso de livros interativos multimodalidade em enfermagem pediátrica nos resultados de aprendizagem do aluno na implementação do programa Merdeka Belajar.

**Métodos:**

estudo de abordagem quase-experimental com um projeto de grupo de controle pós-teste. A amostra incluiu 52 alunos de enfermagem do quarto semestre do Instituto de Ciências da Saúde Suaka Insan Banjarmasin que frequentaram o curso de Enfermagem Pediátrica. Foi utilizada a técnica de amostragem total e o teste t dependente na análise dos dados.

**Resultados:**

o livro interativo multimodalidade de enfermagem pediátrica foi eficaz para melhorar o desempenho de aprendizagem dos alunos do quarto semestre com um valor de significância de 0,015 < 0,05. Há uma diferença de 3,173 no valor médio dos cursos de enfermagem pediátrica da classe A e da classe B.

**Conclusões:**

o uso de livros interativos multimodalidade é muito bom para os professores na aplicação do ensino a fim de ajudar os alunos no entendimento do material que estão aprendendo.

## RESUMEN

**Objetivos:**

analizar la efectividad del uso de libros interactivos multimodales en enfermería pediátrica sobre los resultados de aprendizaje de los estudiantes en la implementación del Programa Merdeka Belajar.

**Métodos:**

investigación cuasi-experimental con un diseño de grupo de control posterior a la prueba. En este estudio, la muestra incluyó 52 estudiantes de enfermería del cuarto semestre del Instituto de Ciencias de la Salud Suaka Insan Banjarmasin que recibieron el curso de Enfermería Pediátrica. Fue utilizada la técnica de muestreo total y la prueba t dependiente en el análisis de datos.

**Resultados:**

el libro interactivo multimodal de enfermería pediátrica fue efectivo en la mejora del logro de aprendizaje de los estudiantes de cuarto semestre con un valor de significancia de 0,015 < 0,05. Hay una diferencia en el valor promedio de los cursos de enfermería pediátrica para la clase A y la clase B, que es de 3,173.

**Conclusiones:**

el uso de libros multimodales interactivos es muy bueno para que los profesores los apliquen en la enseñanza para ayudar a los estudiantes a comprender el material que están aprendiendo.

## INTRODUCTION

The application of Merdeka Belajar integrated with industry 4.0 has three infrastructure requirements, namely: a stable internet network (high speed), digital-based learning instruments and professional and quality teachers or mentors^(1)^. Concern about the digitization of education have become central issues in educational policy decision-making, and as a result, much research in recent years has focused on the development of learning approaches that are globally in demand^(2)^.

Institute of Health Science Suaka Insan is a private university engaged in the field of nursing. This tertiary institution has also implemented an independent learning policy that is integrated with digitization of education. One of the implementations carried out by lecturers in supporting the Merdeka Learning campus is a change in learning methods. Initially, the lecturer provided learning material with the lecture method changing direction Student Centered Learning (SCL). Institute of Health Science Suaka Insan has also utilized digital based learning media for the learning process such as: using video demonstrations to explain laboratory practices and using PowerPoint to help explain material.

Preliminary studies conducted by researchers and the team with personal communication to several nursing lecturers at Institute of Health Science Suaka Insan Banjarmasin said that the books that had been used for the learning process were limited to hardcover in the library or teaching materials such as modules or handouts from PowerPoints, for student reading material. Some lecturers are still not much exposed to the use of technology that can be used as learning media. In addition, based on the results of interviews with several students, they said that sometimes they had difficulty understanding the material because there were too many study materials, even though handouts, modules or book references were given by the lecturer. Lack of interest in reading sources is also one of the reasons they find it difficult to understand the material. The teaching materials given to students are generally a collection of texts taken from several source books. This resulted in the material provided was less varied and tedious, resulting in students not being interested in reading the material.

Based on the discussion above, it can be seen that the link between the implementation of Merdeka Belajar integrated with digitalization in education, namely lecturers are required to be able to improve their abilities in the use of technology, especially in the digital-based learning. Lecturers are required to be able to create digital-based learning media in order to be able to attract student enthusiasm in learning. This is of course a big challenge for lecturers who are not familiar with technology in the learning process.

Apart from that, the nursing course itself has quite a large number of credits in one semester, one of which is the pediatric nursing course. The number of credits for this course namely 4 credits consisting of 2 theoretical credits, 1 credits of laboratory practice and 1 credit of clinical practice according to the higher education curriculum of the Bachelor of Nursing study program Institute of Health Science Suaka Insan Banjarmasin (adjusted to the Indonesian National Qualifications Framework Permen-ristekdikti no.44 of 2015). Student learning outcomes are at the stage of being able to analyze and master nursing care both independently and in groups. Students will certainly experience difficulties in understanding this material if they are only limited to reading the text^(3)^.

Researchers and teams try to develop interactive multimodality books using ebook creator applications. Materials for pediatric nursing course in the Interactive Multimodality Book made by researchers are presented with pictures, text, sound, motion and color videos as well as websites. This Interactive Multimodality Book is expected to be an alternative that can create students interesting to see and read, interactive and not boring so, it becomes the main attraction to make it easier for students to learn material related to Pediatric Nursing.

In line with the opinion above, according to Anggraini suggests that in teaching and learning activities, learning media have a major influence on various senses. Types of audiovisual media (pictures and sound) can increase students’ understanding and interest in the material presented^(4)^. Mayer (2001) argues that student’s learning becomes more meaningful when an array of interactive tools and resources are deployed rather than using text alone. Moreover, the visual representation of content is vital for communicating subject matter and improving students’ understanding^(5)^.

Based on the results of the literature study that the researchers did, there has been no research that examines the Interactive Multimodality Book using the book creator application used in nursing education, especially in pediatric nursing courses. However, there are several similar learning media that have been used before, such as the use of 3D Page Flip Pro digital books and the use of Mobile Assisted Language Learning (MALL).

## OBJECTIVES

To analyze the effectiveness of the use of Interactive Multimodality Books in Pediatric Nursing on Student Learning Outcomes in Implementing the Merdeka Belajar Program.

## METHODS

### Ethical aspects

This research has been conducted an ethical review at the University of Muhammadiyah Banjarmasin.

### Study design, location, and time period

This research is a quasi-experimental study, with a posttest control group design guided by the Quality improvement studies. The research started from 07 March to 09 September 2022 at Institute of Health Science Suaka Insan Banjarmasin.

### Population and sample

The sample in this study, namely 52 students who were divided into 2 groups, namely class A as many as 29 students (control group) and class B as many as 32 students (intervention group). The sampling technique used is Total Sampling. The inclusion criteria are the fourth semester nursing students of Institute of Health Science Suaka Insan Banjarmasin who received the Pediatric Nursing course. Exclusion criteria in this study: nursing students who were absent due to illness or permission for more than 3 days.

### Analysis of results and statistics

The data were analyzed and found if the effect of Using Interactive Multimodality Books in Pediatric Nursing on Student Learning Outcomes was significant p-value <0.05 by the 2-mean dependent difference test (paired sample test).

### Study protocol

The instrument used is an interactive multimodality book on pediatric nursing in the form of an e-book (digital book), measuring nursing student learning achievement, the evaluation instrument used is a student worksheet to see psychomotor aspects (skills), quiz and Google Form (to assess cognitive abilities knowledge) and attitude and performance assessment sheets.

The interactive multimodality book on pediatric nursing made using the book creator application, the steps in making digital textbooks through the book creator application. Make sure the computer or laptop that we will use has Google Chrome installed first. Next point the URL in our Google Chrome address bar to the address https://bookcreator.com/. Furthermore, the researchers and the team began to make textbooks. The textbooks that the researchers and the team created are based on pediatric nursing books related to the latest supporting materials and journals.

**Figure 1:**
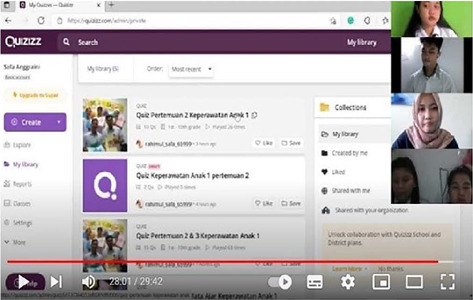
Knowledge assessment

**Figure 2:**
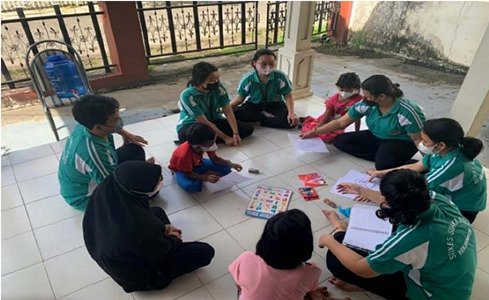
Skill, attitude & performance assessment

In the intervention group, they were given an interactive multimodality textbook for pediatric nursing in the form of an e-book for 4 months to be studied independently. Students in this group were asked to learn using the self-directed learning method.

During the research process data was collected online (using the Quizizz and Google Form) to assess the extent to which students understand the material that has been delivered by lecturers and offline direct observation of student performance where here they apply the material that has been obtained. For example, in the interactive multimodality book on pediatric nursing, there is a topic about playing in healthy children. Students are expected to be able to carry out play therapy in children according to the stages of their growth and development. The last is an assessment of students’ attitudes and performance during teamwork which is measured through an assessment form. The following is a picture of the implementation of the student’s knowledge and skills assessment:

## RESULTS

### Characteristics of respondents based on age and sex

Based on Table 1, it shows the characteristics of the most respondents based on age are the age of 20 years as many as 32 (52%) students, then followed by the age of 19 years as many as 12 (20%) students and 21 years old as many as 11 (18%) students. While the youngest age is at the age of 22 years as many as 3 (5%) students, age 18 years as many as 2 (3%) students and age 25 years as many as 1 (2%) students. The characteristics of the most respondents based on sex, 48 students (78%) were female and the remaining 13 students were male (22%).

**Table 1:** Characterize of respondents

Characterize	n	%
Age		
18 years old	2	3
19 years old	12	20
20 years old	32	52
21 years old	11	18
22 years old	3	5
25 years old	1	2
Sex		
Male	13	22
Female	48	78

Based on Table 2, it shows the average value for pediatric nursing courses fourth-semester students class A is 74.45 while for class B it is 77.62. Thus, statistically descriptive, it can be concluded that there is a difference in the average value of child nursing courses for fourth-semester students based on cognitive, psychomotor and affective aspects between class A and class B.

**Table 2:** Identifying the average value of pediatric nursing courses for fourth semester students based on cognitive, psychomotor and affective

	Class A and B	N	Mean	SD	Std. Error Mean
Value	Class A	29	74.4517	4.78231	0.88805
	Class B	32	77.6250	5.08013	0.89805

Based on Table 3, it shows the value of *Levene’s test for equality of variances* is 0.355 > 0.05, it can be interpreted that the variance the data between class A and class B is homogeneous or the same. The significance value is 0.015 < 0.05. Thus, it can be concluded that the interactive multimodality book on pediatric nursing is effective in increasing the learning achievement of the fourth semester students. Table *mean difference*, it is found that the average difference in the value of pediatric nursing courses for class A with class B is 3.173 where class B who is given an interactive multimodality book on pediatric nursing has a higher average value compared to class A who does not get an interactive multimodality book.

**Table 3:** The effectiveness of the use of interactive multimodality books on learning outcomes of child nursing courses I students of semester IV Class XIV

Description	Levene’s test	Sample t-test		95% CI	
	Sig.	Sig. (2-tailed)	Mean difference	Lower	Upper
Effectiveness of using interactive multimodality book	0.355	0.015	−3.173	−5.708	−.6384

## DISCUSSION

The use of appropriate teaching materials for students is very important because without relevant and contextual teaching materials, learning outcomes will not be optimal. The development of teaching materials is very much needed considering that in learning nursing, students are facilitated with teaching materials that are in accordance with the needs of students in the academic world and the world of work^(6)^. The learning approach using multimodality books is a theory of conveying ideas from reading materials, communication and social semiotics. This approach emphasizes the integration of images, colors, words, and audio as communication media^(7)^. The way multimodality is perceived and enacted is in tandem with how technologies are deployed and used for designing and delivering learning content^(8)^.

Based on the results of the study, the significance value showed 0.015 <0.05. Thus, it can be concluded that the interactive multimodality book on pediatric nursing is effective in improving the learning achievement of fourth semester students. Based on the mean difference, it is found that the average difference in the value of pediatric nursing courses for class A and class B is 3.173 where class B who is given an interactive multimodality nursing book has a higher average value compared to class A who does not get an interactive multimodality nursing book.

According to the researcher, this is because interactive multi-modality books are presented with images, text, sound, motion and color videos as well as websites, where it makes students interested in the appearance of the textbook. This is also in line with the results of research Sesmiyanti, dkk. saying that someone will be interested in reading material if the book displays lots of pictures, sounds and other interesting things that are not only monotonous in writing^(6)^.

Pediatric nursing textbooks made by the research team consisted of ten study materials. Then, this study material was made into interesting materials which consisted of colorful pictures, additional voice explanations from teaching lecturers, and web and links to open sites that directly connected to the material. This was done by the researchers and the team so that students could easily understand the material provided.

Researchers obtained data via google forms which were distributed after students finished attending pediatric nursing lectures related to the use of interactive multimodality e-books, to the intervention group, namely they revealed interactive multimodality books pediatric nursing is very interesting, clear and not boring to read, it really helps them to understand the material because in addition to using interesting animated images, they can also hear the lecturer’s explanation directly through the audio. In addition, because it is in the form of an ebook, it can be opened anytime and anywhere, both offline and online, making it very easy for students to study without being limited by time and place.

Images, text, sound, motion and color videos as well as websites that are included in the Interactive Multimodality Book of pediatric nursing have an important role as an attraction so that students have the intention to read and make it easier for students to understand the material presented. This is in line with the opinion expressed by Weninger, which states that the use of multimodal in a text gives the reader multiple meanings so that the reader can understand and analyze the text based on the meaning of the image and the meaning of the reading^(9)^.

In line with the opinion above, according to Anggraini, suggests that in teaching and learning activities, learning media have a major influence on various senses. Types of audiovisual media (images and sound) are able to increase students’ understanding and interest in the material presented so that learning objectives can be achieved^(4)^. Mayer argues that student’s learning becomes more meaningful when an array of interactive tools and resources are deployed rather than using text alone. Moreover, the visual representation of content is vital for communicating subject matter and improving students’ understanding^(5)^.

In addition, this Interactive Multimodality Book of pediatric nursing also contains several elements such as: motivational elements, enabling elements, assessment elements and reflection elements in each chapter. Each chapter in this book consists of several topics, which begin with learning objectives and providing material to increase student knowledge. Then ends with practice questions. The practice questions in this book aim to assess the extent to which students understand the material that has been studied. This exercise also directs students to apply the knowledge that has been obtained to solve the problems that exist in the Exercise. It also helps students to improve their critical thinking, creative and innovative thinking so that the expected competencies can be achieved. Learning to use the Multimodality Interactive pediatric nursing book allows students to have a different learning experience from previous learning. They can interact with peers, children at different ages and parents of children like they have never done before.

This is in line with the research results of Darmawan, dkk., which state that learning using digital books can improve students’ critical thinking skills. The highest achievement was obtained on the indicator of deciding an action of 68.87%. The results of the study show that overall student learning outcomes have increased^(10)^.

According to Febriyani & Kurniawan, the use of the right media will greatly support success in the learning process. On the other hand, the use of inappropriate media will only waste money and energy, especially for the achievement of learnin objectives which will be far from expectations. One of the learning facilities, universities must be able to provide the right media to support the academic community in learning so that they are not bored in receiving learning in class^(11)^.

Several countries abroad have also implemented learning media using multimodality in the learning process. Teachers from Finland and Denmark (95% and 100% respectively) indicated that they use technology for collaborative learning practices. This may shows that the use of collaborative practices mediated by multimodal technologies is prevalent, as teachers increasingly seem to be confident in integrating collaborative activities that necessitate participatory tools and multiple modes. Participants were also asked to specify the modalities they use to enhance collaborative practices. Collectively the data show that the use of collaborative projects mediated by multimodal technologies was the most prominent choice between the others for the teachers from the three surveyed countries. Formative assessment seemed to be an important part of collaborative teaching strategies since the focus is to help the student understand comprehensively not only how to improve individually but also how the overall performance of the team could be enhanced to communally produce meaningful ideas^(12)^.

### Study limitations

The limitation of this research is that it only involves the dependent variable and the independent variable so that the results obtained cause a bias in it, namely the confounding bias.

### Contributions to the Field

All lecturers are expected to be able to answer global challenges, namely being able to adapt to create various interesting and interactive teaching media to support the learning process so as to increase student interest and motivation to learn. Nursing lecturers in particular, can apply this multimodality book in their courses to help make it easier for students to understand the material given.

## CONCLUSIONS

The interactive multimodality pediatric nursing book is effective for improving the learning achievement of fourth semester students with a significance value of 0.015 < 0,05.
